# Establishment of a Successive Markerless Mutation System in *Haemophilus parasuis* through Natural Transformation

**DOI:** 10.1371/journal.pone.0127393

**Published:** 2015-05-18

**Authors:** Luhua Zhang, Ying Li, Ke Dai, Xintian Wen, Rui Wu, Xiaobo Huang, Jin Jin, Kui Xu, Qigui Yan, Yong Huang, Xiaoping Ma, Yiping Wen, Sanjie Cao

**Affiliations:** Porcine Disease Research Center, College of Veterinary Medicine, Sichuan Agricultural University, Chengdu, 611130, PR China

## Abstract

*Haemophilus parasuis*, belonging to the family *Pasteurellaceae*, is the causative agent of Glässer’s disease leading to serious economic losses. In this study, a successive markerless mutation system for *H*. *parasuis *using two sequential steps of natural transformation was developed. By the first homologous recombination, the target genes were replaced by a cassette carrying kanamycin resistance gene and *sacB* (which confers sensitivity to sucrose) gene using kanamycin selection, followed by the second reconstruction to remove the selection cassette, with application of sucrose to further screen unmarked mutants. To improve DNA transformation frequency, several parameters have been analyzed further in this work. With this method, two unmarked deletions in one strain have been generated successfully. It is demonstrated that this system can be employed to construct multi-gene scarless deletions, which is of great help for developing live attenuated vaccines for *H*. *parasuis*.

## Introduction

*Haemophilus parasuis* (*H*. *parasuis*), a member of the family *Pasteurellaceae*, is the causative agent of Glässer’s disease, which is characterized by polyserositis, arthritis, and meningitis [1]. The infection of *H*. *parasuis* produces significant mortality and morbidity in pig farms, leading to serious economic losses [2]. To explore the pathogenic mechanisms of *H*. *parasuis*, several virulence factors have been identified that may contribute to the Glässer’s disease and it is likely that more virulence determinants will be revealed in the future [3–9]. To date, several complete genome sequences of *H*. *parasuis* have been determined [10–14], therefore the availability of a more satisfactory method for genetic manipulation in *H*. *parasuis* will be of great help to further elucidate the biological function and pathogenesis of target genes.

So far allele replacement systems in *H*. *parasuis* are limited, and the natural transformation system developed by Bigas, *et al*. [15] and modified by Zhang, *et al*. [4] might be the most widely used one. Natural transformation is the process by which bacteria take up DNA from the environment and incorporate it into the host chromosome by homologous recombination [16]. The natural transformation system in *H*. *parasuis* prefers to uptake donor DNA with an uptake signal sequence (USS) of ACCGCTTGT [4]. With this system, several knockout mutants of *H*. *parasuis* have been obtained [3,4,6,7,15,17–19], in which an antibiotic resistance marker was integrated to replace the target gene. The problem is that the expression of other genes may be affected by the antibiotic resistance marker [20], and it is difficult to construct multi-gene mutants with this method, because of the multiple antibiotic resistance markers required [6,18]. Furthermore, due to the presence of antibiotic resistance markers it is not the ideal method to construct live attenuated vaccines for *H*. *parasuis*.

In this study, on the basis of the existing natural transformation methodology [4,15], a simple two-step natural transformation method to construct unmarked mutants in *H*. *parasuis* is present. With this procedure, two genes in *H*. *parasuis* encoding periplasmic serine protease (HtrA) and putrescine/spermidine ABC transporter substrate-binding protein (PotD), identified in our previous research [21], were deleted leaving no antibiotic resistance markers.

## Materials and Methods

### Bacterial strains, plasmids, primers and culture conditions

Bacterial strains and plasmids used in this study are listed in Table 1. PCR primers used in this study (listed in Table 2) were synthesized at Invitrogen (Shanghai, China). *Escherichia coli* DH5α used for plasmid construction and *E*. *coli* BL21 (DE3) used for protein expression were grown in Luria–Bertani (LB) broth or on LB agar. Where necessary, the media were supplemented with 50 μg/mL kanamycin (Kan), 100 μg/mL ampicillin (Amp) or 25 μg/mL chloramphenicol (Cm). *H*. *parasuis* field strains were grown on Tryptic Soy agar (TSA) (Difco Laboratories, Detroit, USA) or in Tryptic Soy Broth medium (TSB) (Difco Laboratories, Detroit, USA) supplemented with 5% bovine serum and 0.01% β-nicotinamide adenine dinucleotide (NAD). When necessary, 20 μg/mL Kan or 2 μg/mL Cm was added for selection of transformants. All strains were grown at 37°C. For counter-selection, *H*. *parasuis* strains were selected on TSA supplemented with 5% bovine serum, 0.01% NAD and 10% sucrose.

**Table 1:** Strains and plasmids used in this study.

Strain or plasmid	Relevant description(s)	Source or reference
Strains		
*Escherichia coli* DH5α	Standard cloning strain	Laboratory collection
*E*. *coli* BL21 (DE3)	Standard expression strain	Laboratory collection
*Haemophilus parasuis*		
MC3	Wild-type strain, non-transformable	Laboratory collection
SC1401	Wild-type strain, transformable	Laboratory collection
SC1402	Wild-type strain, non-transformable	Laboratory collection
SC1403	Wild-type strain, non-transformable	Laboratory collection
SC1404	Wild-type strain, non-transformable	Laboratory collection
SC1405	Wild-type strain, non-transformable	Laboratory collection
SC1401Δ*htrA*::*kan*	SC1401 derivative, contains the *kan* cassette in the *htrA* deletion	This study
SC1401Δ*htrA*::*kan-sacB*	SC1401 derivative, contains the *kan-sacB* cassette in the *htrA* deletion	This study
SC1401Δ*htrA*	SC1401 derivative, *htrA* deletion	This study
SC1401Δ*htrA* Δ*potD*::*kan-sacB*	SC1401 Δ*htrA* derivative, contains the *kan-sacB* cassette in the *potD* deletion	This study
SC1401Δ*htrA* Δ*potD*	SC1401 Δ*htrA* derivative, *potD* deletion	This study
Plasmids		
pET22b	Amp^R^, *E*. *coli* expression vector	Laboratory collection
pET22b-*htrA*	Amp^R^, expression vector carrying *htrA* gene	This study
pET22b-*potD*	Amp^R^, expression vector carrying *potD* gene	This study
pKD3	Cm^R^, chloramphenicol resistance cassette-carrying vector	[25]
pKD4	Kan^R^, kanamycin resistance cassette-carrying vector	[25]
pMD19-T	Amp^R^, *E*. *coli* cloning vector	Takara
pK18mobsacB	Kan^R^, suicide and narrow-broad-host vector	[31]
pMDHK	Amp^R^, Kan^R^, a 2153-bp fragment containing the motif of 5'-ACCGCTTGT and the Δ*htrA*::*kan* cassette in pMD19-T	This study
pKBHK	Kan^R^, a 2153-bp fragment containing the motif of 5'-ACCGCTTGT and the Δ*htrA*::*kan* cassette in pK18mobsacB	This study
pMDHKS	Amp^R^, Kan^R^, a 4348-bp fragment containing the motif of 5'-ACCGCTTGT and the Δ*htrA*::*kan-sacB* cassette in pMD19-T	This study
pMDH	Amp^R^, a 1218-bp fragment containing the motif of 5'-ACCGCTTGT and the Δ*htrA* cassette in pMD19-T	This study
pMDPKS	Amp^R^, Kan^R^, a 4348-bp fragment containing the motif of 5'-ACCGCTTGT and the Δ*potD*::*kan-sacB* cassette in pMD19-T	This study
pMDP	Amp^R^, a 1218-bp fragment containing the motif of 5'-ACCGCTTGT and the Δ*potD* cassette in pMD19-T	This study

**Table 2:** Primers used in this study.

Primer name	Primer sequence (5'→3')
P1	**CATGCCTGCAGGTCGACGAT** ACCGCTTGTCACAGAAGTAGTCGTGTACT
P2	**GCAGGGCTTCCCAACCTTAC**TGATTATCCTTACTAAATTC
P3	**GGGGTTCGAAATGACCGACC**AAAGTTTTGCGATACTATCA
P4	**CCCGGGGATCCTCTAGAGAT** ACCGCTTGTTTTTGTTCAATCGGTGCATT
P5	GTAAGGTTGGGAAGCCCTGC
P6	GGTCGGTCATTTCGAACCCC
P7	CGTAATACGACTCACTATAG
P8	GTTCCGCTTCCTTTAGCAG
P9	**CTATAGTGAGTCGTATTACG**GGTCGGTCATTTCGAACCCC
P10	**CTGCTAAAGGAAGCGGAAC**AAAGTTTTGCGATACTATCATT
P11	**TGATAGTATCGCAAAACTTT**TGATTATCCTTACTAAATTC
P12	AAAGTTTTGCGATACTATCA
P13	**CATGCCTGCAGGTCGACGAT** ACCGCTTGTCATTATTGATTTTATTGATA
P14	**TTTAAGCGATGTCTATGGAA**CAATGTATTCTCCTAAAAGA
P15	TTCCATAGACATCGCTTAAA
P16	**CCCGGGGATCCTCTAGAGAT** ACCGCTTGTTGCCTGTTGATTTCACCATA
P17	**GCAGGGCTTCCCAACCTTAC**CAATGTATTCTCCTAAAAGA
P18	**CTGCTAAAGGAAGCGGAAC**TTCCATAGACATCGCTTAAA
P19	CATG*CCATGG*CATTGCCTACTGCTGTAAACGG
P20	CCG*CTCGAG*ATTAATGATTACATAGAAAT

The 20-bp extensions required for In-Fusion cloning are indicated in bold text. The USS of *H*. *parasuis* is underlined. NcoI and XhoI sites in the primers P19 and P20 are indicated in italics.

### DNA manipulations

Genomic DNA extractions were performed using TIANamp Bacteria DNA Kit (Tiangen, China), plasmid DNA extractions were performed using Plasmid Mini Kit (Omega, USA), and PCRs were performed with either PrimeSTAR Max Premix (Takara, Japan) or Phanta Super-Fidelity DNA Polymerase mix (Vazyme, China) according to the manufacturers’ protocols. PCR fragments were obtained from PCR mixtures and purified by agarose gel electrophoresis using Biowest Regular Agarose (Biowest, Spain) and Gel Extraction Kit (Omega, USA). Restriction enzymes were purchased from Takara. DNA concentrations were measured by SmartSpec Plus (Biorad, USA).

### Construction of plasmid pMDHK

**Fig 1:**
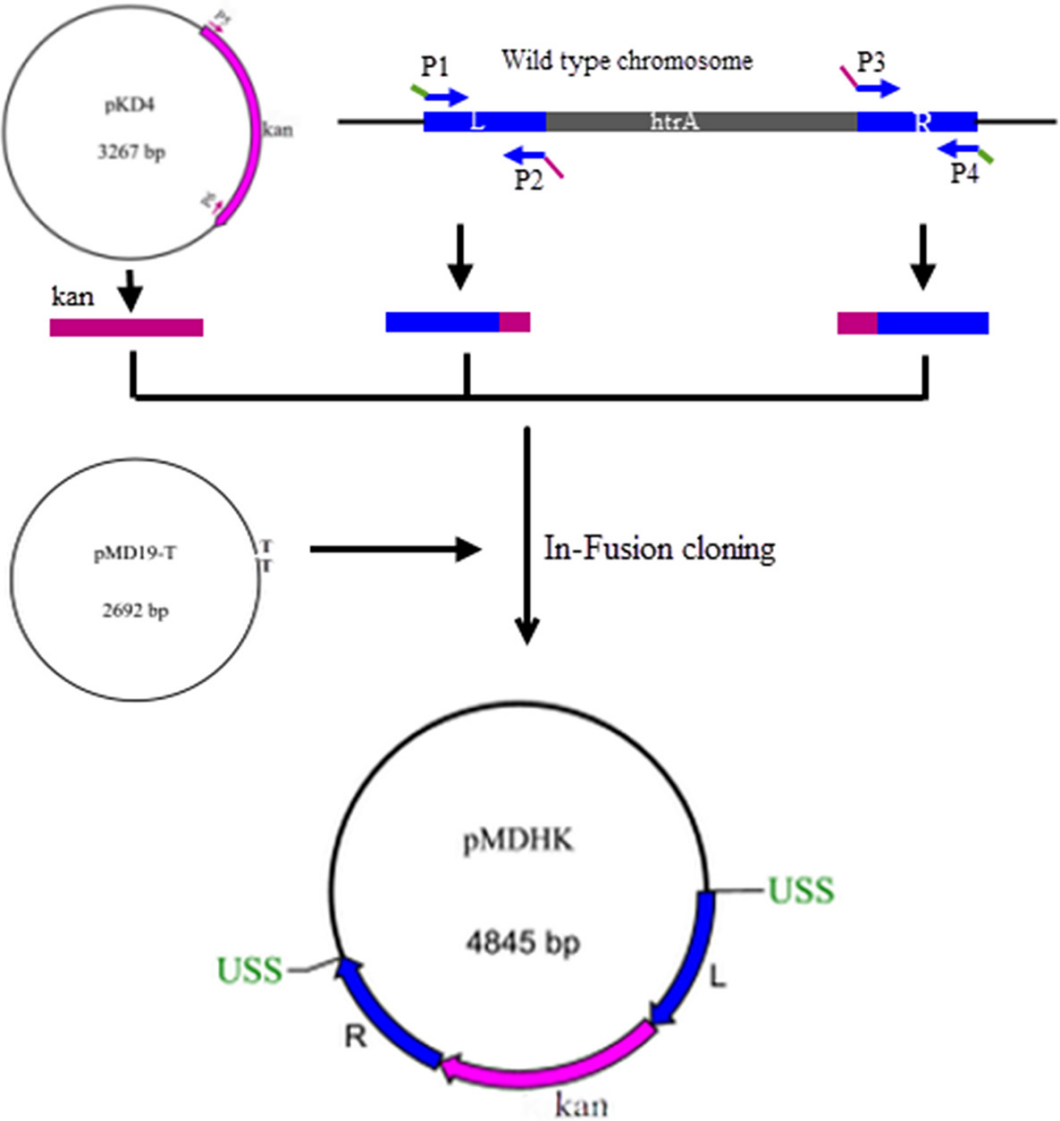
Scheme for the construction of plasmid pMDHK. Primer pairs P1/P2 and P3/P4 were used to amplify the upstream and downstream regions of the *htrA* gene respectively, each with 20-bp end sequences identical to the *kan* fragment (using primer P2 and P3) and 20-bp end sequences identical to vector pMD19-T (using primer P1 and P4). Primers P5/P6 were used to amplify the *kan* fragment from the plasmid pKD4. The resulting three PCR products and the cloning vector pMD19-T were joined by In-Fusion cloning. Two USSs required for efficient transformation were introduced into the plasmid pMDHK by using primer P1 and P4.

For lacking the available marked genomic DNA, a marked plasmid pMDHK was constructed to identify some possible transformable strains of *H*. *parasuis*. First of all, genomic DNA of *H*. *parasuis* isolate MC3 was used as a template to amplify the 600-bp upstream and downstream regions flanking the *htrA* gene using primers P1 and P2 and primers P3 and P4. Both DNA fragments contained the 9-bp DNA uptake signal sequence (USS) of 5'-ACCGCTTGT [22]. In parallel, the kanamycin resistance cassette (*kan*) was amplified from a pKD4 plasmid with primers P5 and P6. The three PCR fragments were mixed with pMD19-T vector (Takara, Japan) and ligated using ClonExpress MultiS One Step Cloning Kit (Vazyme, China) according to the manufacturer’s protocol. The resulting products were transformed into *E*. *coli* DH5α and transformants were selected on LB agar containing 50 μg/mL Kan. PCRs were performed on selected colonies to confirm the presence of inserts. The resulting plasmid pMDHK (Fig 1) was used to transform *H*. *parasuis* isolates for screening possible competent cells as described below.

### Screening transformable strain(s)

Prior to performing transformation experiments, the level of spontaneous resistance to Kan was evaluated by plating middle exponential phase cultures of *H*. *parasuis* isolates onto selective and non-selective TSA plate.

The transformation experiments were carried out as previously described [4] with some modifications. Briefly, a single colony was transferred from a TSA plate into 5 mL TSB for cultivation at 37°C overnight. Then, 50 μL of bacteria were spotted onto a TSA plate and spread in a small area. After 24 h of incubation at 37°C, the bacteria were scraped up and resuspended in 50 μL TSB, 20 μL of which were mixed with cAMP to a concentration of 8 mM and incubated at room temperature. After 10 min of incubation, the cells were mixed with 1 μg donor plasmid pMDHK and spotted onto a TSA plate and spread in a small area. After 5 h of incubation at 37°C, bacteria were scraped up and plated onto selective and non-selective TSA plate for cultivation. Instead of donor plasmid, TE buffer was added and mixed with the cells as a negative control. PCRs were employed to confirm that colonies on selective TSA plates were true transformants. Transformation frequency was measured by the number of antibiotic resistant cfu per mL recovered divided by the total cfu per mL counted on non-selective TSA plate. The transformable strains identified were used for further study.

### Construction of plasmid pMDHKS

**Fig 2:**
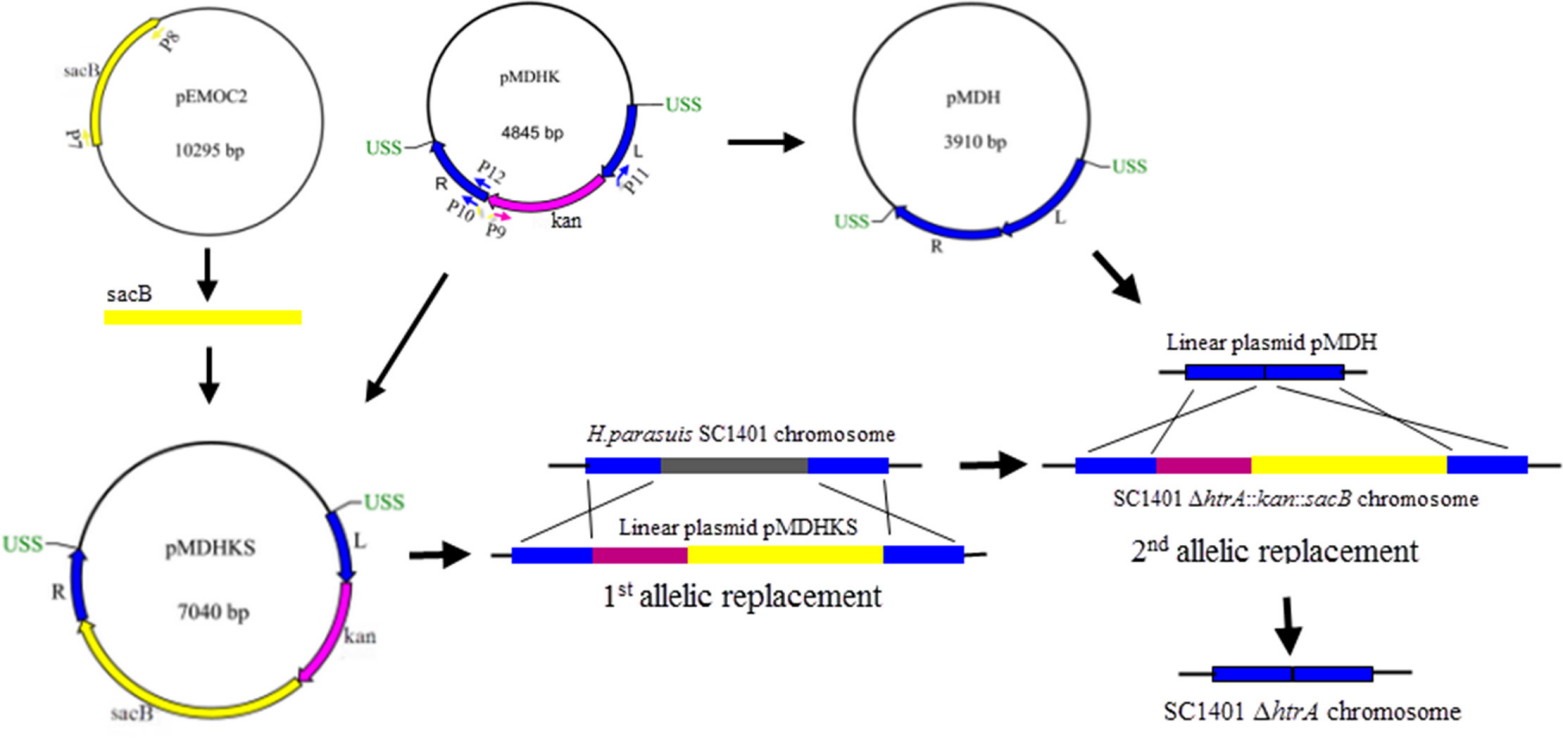
Scheme for the construction of the unmarked SC1401 Δ*htrA* strain. The plasmid pMDHKS was generated by inserting the *sacB* fragment (amplified from the plasmid pEMOC2 using primer pairs P7/P8) into the plasmid pMDHK by inverse PCR (using primer P9 and P10) and In-Fusion cloning. The plasmid pMDH was obtained by inverse PCR (using primer P11 and P12) and In-Fusion cloning. To construct the unmarked SC1401 *ΔhtrA* strain, the *kan-sacB* cassette was integrated into the SC1401 chromosome and the *htrA* gene was replaced by the first allelic replacement. Next, the linearized plasmid pMDH was employed in the second allelic replacement to remove the *kan-sacB* cassette from the SC1401Δ*htrA*::*kan*-*sacB* chromosome, leaving an unmarked *htrA* deletion strain.

Plasmid pMDHKS (Fig 2) was constructed for *htrA* gene deletion and counter-selection in *H*. *parasuis* transformable strains. To construct this plasmid, the *sacB* gene was initially amplified from pEMOC2 [23] by PCR using primers P7 and P8. Then inverse PCR was employed to open up the plasmid pMDHK with primers P9 and P10, adding 20 bp extensions to allow insertion of *sacB* gene by In-Fusion cloning. The two PCR products were purified by agarose gel electrophoresis using Biowest Regular Agarose (Biowest, Spain) and harvested using Gel Extraction Kit (Omega, USA). The two purified fragments were mixed and ligated using ClonExpress MultiS One Step Cloning Kit (Vazyme, China) according to the manufacturer’s protocol. The resulting products were transformed into *E*. *coli* DH5α and transformants were selected on LB-Kan. PCR was employed to confirm the presence of *sacB* gene. The sucrose sensitivity of selected transformants was detected by plating on LB agar supplemented with 10% sucrose.

### Construction of plasmid pMDH

Plasmid pMDH (Fig 2) was constructed to remove the *kan* and *sacB* genes from the genomic DNA of insertion-deletion mutants in the second transformation step. The inverse PCR was carried out to open up the plasmid pMDHK with primers P11 and P12, removing the *kan* gene and adding 20 bp overhangs to allow direct fusion by In-Fusion cloning with ClonExpress MultiS One Step Cloning Kit (Vazyme, China). The reaction mixture was transformed into *E*. *coli* DH5α and transformants were selected on LB agar supplemented with 100 mg/mL Amp.

### Construction of an unmarked Δ*htrA* mutant of *H*. *parasuis* SC1401

An unmarked *htrA* mutant was constructed using a two-step natural transformation method (Fig 2). First, the plasmid pMDHKS was linearised with EcoRI and used to transform *H*. *parasuis* SC1401 using natural transformation method as described above. PCRs were employed to confirm the appropriate insertion-deletion in Kan-resistant colonies, and the true transformants were detected for the sucrose sensitivity by plating on TSA supplemented with 10% sucrose. Second, the plasmid pMDH was also linearised with EcoRI and transformed into the true transformants generated in the first step to delete the *kan-sacB* cassette. Transformants were screened on TSA supplemented with 10% sucrose and on TSA supplemented with 20 μg/mL Kan. PCRs were carried out to confirm the appropriate deletion in colonies resistant to sucrose and sensitive to Kan.

### Construction of unmarked Δ*htrA* Δ*potD* mutant of *H*. *parasuis* SC1401

To delete *potD* gene from genome of *H*. *parasuis* SC1401 Δ*htrA*, two plasmids pMDP and pMDPKS were constructed. First, the upstream and downstream regions flanking the *potD* gene were amplified from genomic DNA of *H*. *parasuis* isolate MC3 using primers P13/P14 and primers P15/P16. Both DNA fragments were added with 9-bp DNA USS [22]. The two PCR fragments were ligated with pMD19-T vector using ClonExpress MultiS One Step Cloning Kit to form pMDP. Transformants were selected on LB-Amp and confirmed by PCRs. Subsequently, inverse PCR was employed to open up the plasmid pMDP with primers P17 and P18, adding 20 bp extensions to allow insertion of *kan*-*sacB* cassette by In-Fusion cloning. In parallel, the *kan*-*sacB* cassette was amplified from plasmid pMDHKS with primers P5 and P8. The two fragments were mixed and ligated using ClonExpress MultiS One Step Cloning Kit to form pMDPKS.

The generation of an unmarked Δ*htrA* Δ*potD* double mutant was performed with the two-step natural transformation method by transformation of the SC1401 Δ*htrA* mutant. First, the plasmid pMDPKS was linearised with EcoRI and used to transform SC1401 Δ*htrA* mutant by natural transformation method as described above. PCRs were employed to confirm the appropriate insertion-deletion in Kan-resistant colonies, and the true transformants were detected for sucrose sensitivity by plating on TSA supplemented with 10% sucrose. Second, the plasmid pMDP was also linearised with EcoRI and transformed into the true transformants generated in the first step to delete the *kan-sacB* cassette. Transformants were screened on TSA supplemented with 10% sucrose and on TSA supplemented with 20 μg/mL Kan. PCRs were carried out to confirm the appropriate deletion in colonies resistant to sucrose and sensitive to Kan.

### Generation of recombinant proteins and antisera and confirmation of mutants by western blotting

The *htrA* gene was amplified from genome of *H*. *parasuis* SC1401 with primers P19 and P20 and cloned into the NcoI and XhoI sites of pET22b to form plasmid pET22b-*htrA*, which was expressed in *E*. *coli* BL21 (DE3). The recombinant HtrA was purified by metal affinity chromatography using Profinity IMAC Ni-Charged Resin (biorad) according to the manufacturer’s protocol. The generation and purification of recombinant PotD were also performed as described above. The production of rabbit antisera against recombinant HtrA and PotD was performed as described [24].

For western blotting analysis, the whole-cell extract of parental strain and mutant strains was analyzed by 12% SDS-PAGE and electrotransferred to a nitrocellulose membrane. After being blocked with 5% nonfat milk in PBST (phosphate-buffered saline containing 0.05% Tween 20) at room temperature (RT) for 30 min, the membrane was incubated at RT for 1h with rabbit antiserum against recombinant HtrA or PotD produced on the above as the primary antibody. Horseradish peroxidase-conjugated goat anti-rabbit IgG (Bioss, China) were used as the secondary antibody. The membrane was developed with Immun-Star Western C Kit (biorad, USA) according to the manufacturer’s instructions.

## Results

### Identification of a transformable strain in *H*. *parasuis*

To identify possible transformable strains, chloramphenicol and kanamycin resistance cassettes from pKD3 and pKD4 [25] were selected as markers for natural transformation. The results showed that no transformants were obtained on TSA-Cm when the isolates were transformed with the plasmid pMDHC, which is identical to the plasmid pMDHK except for the resistance marker. One isolate SC1401 among the six screened strains was identified as a highly transformable strain on TSA-Kan using plasmid pMDHK.

### Optimized conditions for DNA uptake in *H*. *parasuis*

To improve DNA transformation of *H*. *parasuis*, several possible parameters were analyzed. First, two USSs required for natural transformation that are present in plasmid pMDHK were based on the report by Zhang, *et al*. [4] but not Bigas, *et al*. [15] as explained earlier [10]. Second, to determine the effect of cAMP in the transformation experiments, the transformable strain SC1401 was transformed with circular plasmid pMDHK in the presence of 8 mM or no cAMP. The data showed no significant difference in the transformation assay (data not shown) and confirmed the previous report [4]. As to the disagreement with the results of Bigas, *et al*. [15], it was postulated that this might be due to a difference in the sensitivity to exogenous cAMP in different strains. Third, in previous researches, vector pK18mobsacB is widely used for natural transformation of *H*. *parasuis* [4,6,7,9,18]. In this study, pMD19-T was employed to construct recombinant suicide plasmids for natural transformation in *H*. *parasuis*. The result showed that mutant SC1401Δ*htrA*::*kan* was constructed successfully on the basis of pMD19-T with a transformation frequency of 7.1 ×10^–4^. Fourth, to explore whether the linear fragments are more likely to promote natural transformation, the transformation efficiency obtained using 1 μg linear plasmid pMDHK was compared to that obtained by 1 μg intact pMDHK. The results showed that the transformants generated by linear plasmid pMDHK increased by 6–15 cfu (data not shown). Furthermore, transformants could be obtained at a low amount of linear plasmid (0.01μg) and no saturation of transformation was found when the amount of linear plasmid was comprised of 0.01μg to 1 μg. It is recommended that 1 μg of donor DNA was adequate for routine transformation. At last, the length of flanking homology for allelic exchange, as shown by previous researches, ranges from 400 to 700 bp [4,6,7,18]. 600-bp homology fragments were employed in our new protocol, in which a transformation frequency of 7.1 ×10^–4^ was achieved.

### A new unmarked deletion in *H*. *parasuis*

To avoid introducing antibiotic resistance genes in mutants, a new two-step natural transformation mutagenesis using the sucrose counterselection was developed. Two recombinant plasmids (Fig 2) were constructed: pMDHKS, which carries the *kan* marker and the *sacB* counterselection marker, and pMDH, which carried no selection marker but only flanking fragments. The procedure of the markerless deletion of *htrA* gene with linear recombinant plasmids is shown in Fig 2. By the first homologous recombination, the *kan*-*sacB* cassette was integrated into the two flanking regions of *htrA* gene on the chromosome seamlessly, so that the original *htrA* gene was replaced by the selection cassette in the resultant mutant. Kan positive selection was highly successful, and the *kan-sacB* cassette was detected in all Kan-resistant clones tested. In the second round of reconstruction, the *kan-sacB* cassette was removed, followed by sucrose counterselection. The sucrose selection was not always stringent for the widespread existence of spontaneous resistance to sucrose. Transformants isolation was achieved for the high transformation frequency of SC1401. 25 colonies on TSA-sucrose were selected and tested for the deletion of *kan-sacB* cassette by PCR, and one was confirmed to yield the appropriate deletion.

### Construction of an unmarked Δ*htrA* Δ*potD* mutant

**Fig 3:**
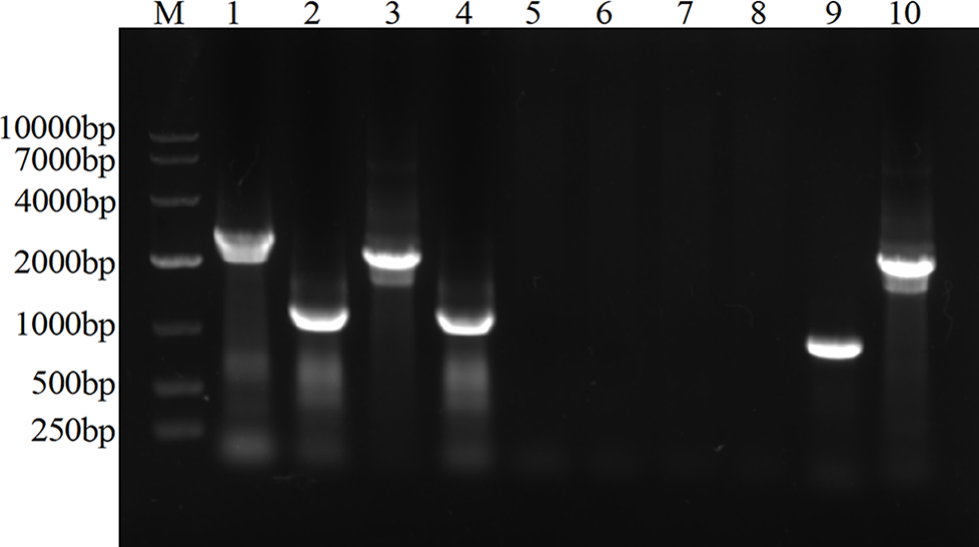
PCR confirmation of the SC1401 Δ*htrA* Δ*potD* strain. PCR amplification was performed with templates of SC1401 genome (lanes 1, 3, 5, 7) and of SC1401 Δ*htrA* Δ*potD* chromosomal DNA (lanes 2, 4, 6, 8) using primers: P1/P4 (lanes 1, 2); P13/P16 (lanes 3, 4); P5/P6 (lanes 5, 6); P7/P8 (lanes 7, 8). Lane 9, PCR fragments were amplified using primers P5/P6 with template of plasmid pKD4 (positive control for *kan* gene). Lane 10, PCR fragments were amplified using primers P7/P8 with template of plasmid pEMOC2 (positive control for *sacB* gene). Lane M, DNA molecular weight markers.

To determine whether this new transformation system could be applied to construct successive markerless deletions in one strain, *potD*, a non-essential gene for growth, was chosen as the target for deletion. 600-bp upstream and downstream regions flanking the *potD* gene were employed to construct plasmids pMDPKS and pMDP. As described above, the appropriate insertion-deletion mutants were obtained easily through the first transformation. More than 60 colonies on TSA-sucrose were screened for the deletion of *kan-sacB* cassette by PCR after the second step, of which one unmarked Δ*htrA* Δ*potD* mutant was finally identified (Fig 3).

### Confirmation of mutants by western blotting

**Fig 4:**
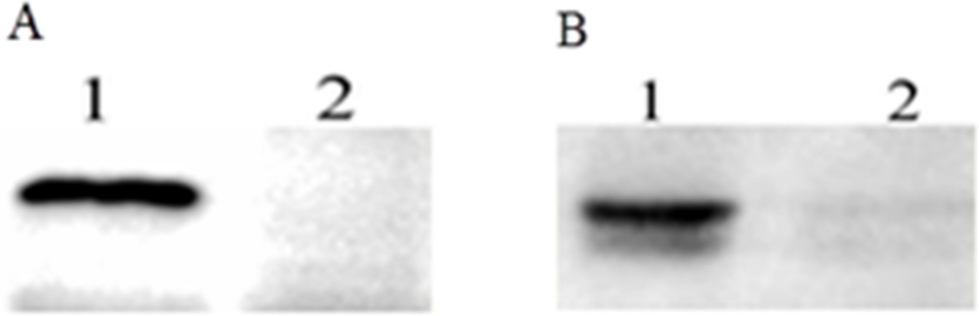
Western blotting analyses of the SC1401 Δ*htrA* Δ*potD* strain. (A) The lysates of the SC1401 (lane 1) and SC1401Δ*htrA* Δ*potD* strain (lane 2) were analyzed using anti-rHtrA antibodies. The wild strain displayed the expected specific band while no band detected accordingly in the Δ*htrA* Δ*potD* strain. (B) The lysates of the SC1401 (lane 1) and SC1401Δ*htrA* Δ*potD* strain (lane 2) were analyzed using anti-rPotD antibodies. The expected specific band was detected in the wild strain but not in the Δ*htrA* Δ*potD* strain.

Western blotting analysis showed that the HtrA and PotD could be detected in the whole-cell extract of wild strain SC1401, while not in the double mutant strain (Fig 4). The results are further evidence for the deletion of *htrA* and *potD* genes in strain SC1401.

## Discussion

In the present work, a simple and highly efficient genetic manipulation system on the basis of natural transformation was developed and successfully introduced two consecutive unmarked deletions into the chromosome of *H*. *parasuis*. The deletions are carried out by two sequential transformation steps, of which the target genes are replaced by *kan-sacB* cassette using Kan selection, followed by the second reconstruction to remove the selection cassette. In this study, counterselection marker *sacB*, which confers sucrose sensitivity, is applied to screen unmarked mutants further. Although spontaneous resistance to sucrose is widespread, high transformable efficiency of clinical isolate SC1401 allows isolation of double crossover mutants to be achieved.

In this method, recombinant plasmids were linearised before transformation to make sure the allele exchange between plasmid and genome was by double-crossover [26]. Two USSs that required for DNA uptake in *H*. *parasuis* were introduced into the recombinant plasmids to promote the exogenous DNA to be internalized. 600-bp flanking homology fragments on each side of the selection cassette meet the needs of efficient double recombination and ensure the reliable amplification by PCR. Unlike previous studies [4,15], the widely used vector pMD19-T was employed to construct recombinant suicide plasmids in this system, by which the markerless mutation system of *H*. *parasuis* can be performed in fewer restrictions. It was suggested that the main function of plasmid DNA is to protect the flanking homologous DNA from degradation by restriction systems [27]. And we propose that PCR fragments containing selection cassettes flanked by homologous sequences on each side could work, of which further identifications are needed.

Vaccination is generally considered as an effective means of controlling infectious diseases. Traditional commercial bacterin of *H*. *parasuis* generally provide strong homologous protection, and none has offered complete satisfaction against challenge with all serovars [28,29]. Recently, Brockmeier et al [30] demonstrated that a live strain could induce cross-protection against heterologous serotypes but further attenuation is required if it would be developed as a live vaccine. Therefore, the establishment of an unmarked knockout method will be of great help. To date, the traditional one-step natural transformation method has been used widely for gene deletions in *H*. *parasuis* with high efficiency but leaving antibiotic resistance markers, which is not permitted for licensing of live attenuated vaccines. Therefore, with the availability of a markerless mutation system developed in this study in *H*. *parasuis*, it is now possible to construct an effective and live vaccine. And for biosafety purposes, multi-gene deletions carrying no antibiotic markers could be achieved with this system to attenuate to a greater extent.

Results in this work have demonstrated that it is possible to generate successive unmarked mutation using the same selectable marker with this methodology in *H*. *parasuis*. The development of an unmarked mutation system in *H*. *parasuis* represents a substantial improvement of genetic manipulation in functional genomics studies and vaccine strategies. This system could be potentially applied to many other Gram-negative bacteria for genetic engineering as a useful tool.
